# Room-temperature neutron and X-ray data collection of 3CL M^pro^ from SARS-CoV-2

**DOI:** 10.1107/S2053230X20011814

**Published:** 2020-09-15

**Authors:** Daniel W. Kneller, Gwyndalyn Phillips, Andrey Kovalevsky, Leighton Coates

**Affiliations:** a Neutron Scattering Division, Oak Ridge National Laboratory, 1 Bethel Valley Road, Oak Ridge, TN 37831, USA; b National Virtual Biotechnology Laboratory, US Department of Energy, USA; c Second Target Station, Oak Ridge National Laboratory, 1 Bethel Valley Road, Oak Ridge, TN 37830, USA

**Keywords:** neutron diffraction, X-ray diffraction, SARS-CoV-2, 3CL M^pro^

## Abstract

The replication of SARS-CoV-2 produces two large polyproteins, pp1a and pp1ab, that are inactive until cleavage by the viral chymotrypsin-like cysteine protease enzyme (3CL M^pro^) into a series of smaller functional proteins. At the heart of 3CL M^pro^ is an unusual catalytic dyad formed by the side chains of His41 and Cys145 and a coordinated water molecule. The catalytic mechanism by which the enzyme operates is still unknown, as crucial information on the protonation states within the active site is unclear. To experimentally determine the protonation states of the catalytic site and of the other residues in the substrate-binding cavity, and to visualize the hydrogen-bonding networks throughout the enzyme, room-temperature neutron and X-ray data were collected from a large H/D-exchanged crystal of ligand-free (apo) 3CL M^pro^.

## Abstract 2

Protein crystallization of 3CL M^pro^ from SARS-CoV-2 is described along with neutron and X-ray data collection.

## 1. Introduction   

COVID-19 is a global pandemic that is causing worldwide disruption to travel, economic activity and social life (Zhou *et al.*, 2020 ▸; Wu *et al.*, 2020*a* ▸,*b* ▸; Coronaviridae Study Group of the International Committee on Taxonomy of Viruses, 2020 ▸). It is caused by the SARS-CoV-2 coronavirus, an enveloped positive-sense single-stranded RNA virus with a genome of around 30 kb. The virus is composed of around 28 proteins that can be broken down into two main classes: nonstructural proteins (NSPs) that are typically involved in viral replication and structural proteins that are involved in forming the structure of the virus. NSP5 or 3CL M^pro^ is a cysteine protease that is essential for viral replication. It performs a critical function by cleaving the two large viral polyproteins pp1a and pp1ab into smaller functional proteins (Gorbalenya & Snijder, 1996 ▸). The lack of a homologous human protein makes 3CL M^pro^ one of the most promising targets for the design of specific protease inhibitors (Zhang *et al.*, 2020 ▸; Kneller *et al.*, 2020 ▸; Jin *et al.*, 2020 ▸). The global crystallographic community has rapidly been exploring chemical space in the search for drug candidates that can block the action of 3CL M^pro^ (Zhang *et al.*, 2020 ▸). To date, no FDA-approved inhibitors (Pillaiyar *et al.*, 2016 ▸) have been developed against the closely related 3CL M^pro^ from the 2003 SARS-CoV that shares 96% amino-acid homology with the 3CL M^pro^ from SARS-CoV-2.

**Figure 1:**
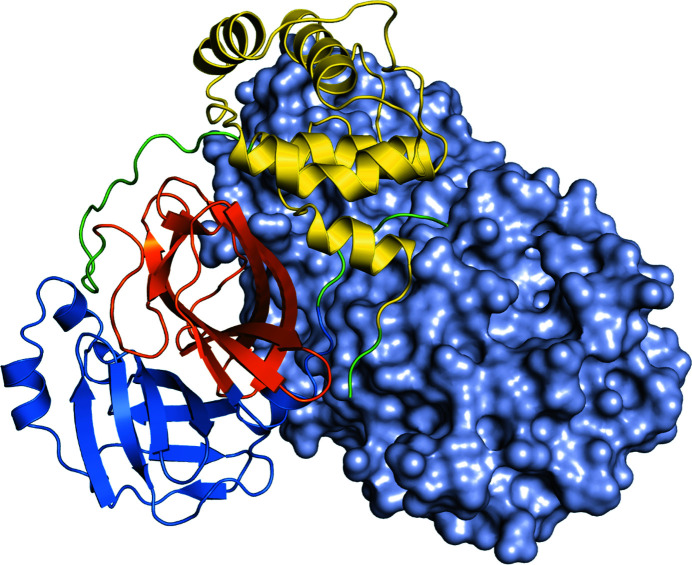
The structure of 3CL M^pro^ from SARS-CoV-2. One polypeptide chain of the dimer is shown as a cartoon, where domain I is colored blue, domain II is colored orange and domain III is colored yellow, whereas the loops connecting the domains are colored green. The other polypeptide chain is shown as a light blue surface.

3CL M^pro^ is active as a homodimer that is composed of two amino-acid chains of 306 amino acids (Kneller *et al.*, 2020 ▸; Zhou *et al.*, 2020 ▸; Jin *et al.*, 2020 ▸). Each chain is folded into three domains. Domain I is composed of residues 8–101, while domain II is composed of residues 102–184. Domains I and II are formed from antiparallel β-barrel structures and are the catalytic domains. Domain III is formed from residues 201–303 and consists mainly of five α-helices where the two polypeptide chains interact to form a dimer (Fig. 1 ▸). Based on earlier studies of SARS-CoV 3CL M^pro^, domain III plays an essential role in the protease function as the monomeric enzyme is not catalytically active (Hsu *et al.*, 2005 ▸).

To improve the drug design of SARS-CoV-2 3CL M^pro^-specific protease inhibitors, knowledge of the complete enzyme structure, including the locations of H atoms, and the direct visualization of hydrogen-bonding networks is of paramount importance. The presence or absence of H atoms on ionizable chemical groups changes the electrical charges of amino-acid side chains and therefore can modulate the electrostatics of the drug-binding cavity. The only technique that is capable of the direct determination of H-atom positions is neutron crystallography. Neutron protein crystallography (NPC) can provide the locations of H and D atoms experimentally rather than by inference based on general chemical knowledge. The atomic nucleus scatters neutrons, and those scattered from deuterium, an isotope of hydrogen, are scattered with the same magnitude as the heavier elements of the protein such as carbon, nitrogen and oxygen. Thus, with NPC the positions of D atoms can be determined directly, even at moderate resolutions of around 2 Å (Bacik *et al.*, 2017 ▸; Vandavasi *et al.*, 2016 ▸; Kovalevsky *et al.*, 2010 ▸; Gerlits *et al.*, 2016 ▸; Kumar *et al.*, 2020 ▸), compared with X-rays, which typically require much higher resolutions of below 1 Å to begin to observe some H-atom positions (Erskine *et al.*, 2003 ▸; Coates *et al.*, 2001 ▸).

Here, we report the crystallization of, and X-ray and neutron diffraction data collection from, a large SARS-CoV-2 3CL M^pro^ crystal of 0.3 mm^3^ in volume. We demonstrate that neutron diffraction-quality crystals of this enzyme can be grown successfully using a microseeding crystallization technique. The crystal of hydrogenous H/D-exchanged 3CL M^pro^ diffracted X-rays to a resolution of 2.3 Å and neutrons to a resolution of 2.5 Å at room temperature.

## 2. Materials and methods   

### 2.1. 3CL M^pro^ expression and purification   

The 3CL M^pro^ (NSP5 M^pro^) gene from SARS-CoV-2 was cloned into the pD451-SR plasmid harboring kanamycin resistance (ATUM, Newark, California, USA). The 3CL M^pro^ gene was surrounded on either side by an upstream maltose-binding protein (MBP) and a downstream His6 tag. This plasmid was transformed into *Escherichia coli* BL21(DE3) cells and sequence-verified. MBP fusion is normally used to improve the solubility of proteins during overexpression, and kanamycin resistance is the preferred selectable marker for future expression in deuterated media. In this construct, MBP is followed by the the SARS-CoV-2 NSP4-NSP5 autoprocessing site SAVLQ↓SGFRK and 3CL M^pro^ is followed by the human rhinovirus 3C protease (HRV 3C) cleavage site SGVTFQ↓GP. MBP and the His_6_ tag can be cleaved by M^pro^ *in vivo* autoprocessing and by *in vitro* HRV 3C protease treatment, respectively, allowing clarification of the authentic 3CL M^pro^ by nickel-affinity chromatography.

Expression of 3CL M^pro^ was performed in Luria–Bertani broth with 1 g l^−1^ glucose using the antibiotic kanamycin (50 mg l^−1^). When the culture had grown to an OD_600_ of 0.8 at 37°C, overexpression was induced with 0.2 m*M* isopropyl β-d-1-thiogalactopyranoside for 18 h at 18°C. The harvested cells were lysed by sonication in 20 m*M* Tris pH 8.0, 150 m*M* NaCl, 40 m*M* imidazole, 1 m*M* tris(2-carboxyethyl)phosphine (TCEP) and centrifuged at 30 000*g* for 30 min. The soluble lysate was loaded onto a HisTrap FF column and eluted as a single peak using a linear gradient of 20 m*M* Tris pH 8.0, 150 m*M* NaCl, 500 m*M* imidazole, 1 m*M* TCEP. The His_6_ tag was removed by treating the pooled eluate with HRV 3C protease also containing a His_6_ tag (Sigma–Aldrich, St Louis, Missouri, USA) at a ratio of 500 mg 3CL M^pro^ to 1 mg HRV 3C protease. The sample was then dialyzed in 20 m*M* Tris pH 8.0, 150 m*M* NaCl, 1 m*M* TCEP for 18 h at 4°C. When this mixture was applied onto a HisTrap FF column, HRV 3C, uncleaved 3CL M^pro^ and cleaved His_6_ tags were retained and authentic 3CL M^pro^ was collected as the flowthrough. The final production yield of purified protein was ∼15 mg per litre of expression culture. The protein was kept in 20 m*M* Tris pH 8.0, 150 m*M* NaCl and fresh 1 m*M* TCEP baseline buffer conditions. Each 3CL M^pro^ molecule features 12 cysteine residues, including the catalytic Cys145, which may be prone to oxidation in the absence of a reducing agent such as TCEP. Protein samples were concentrated to 4–6 mg ml^−1^ (∼120–180 µ*M* based on monomers) for crystallization using polyethersulfone membrane centrifugal concentrators (Sartorius, Göttingen, Germany). M^pro^ is expected to be primarily dimeric at this concentration as the homodimer dissociation constant has previously been measured to be 2.5 µ*M* by analytical ultracentrifugation (Zhang *et al.*, 2020 ▸).

### 2.2. 3CL M^pro^ crystallization   

**Figure 2:**
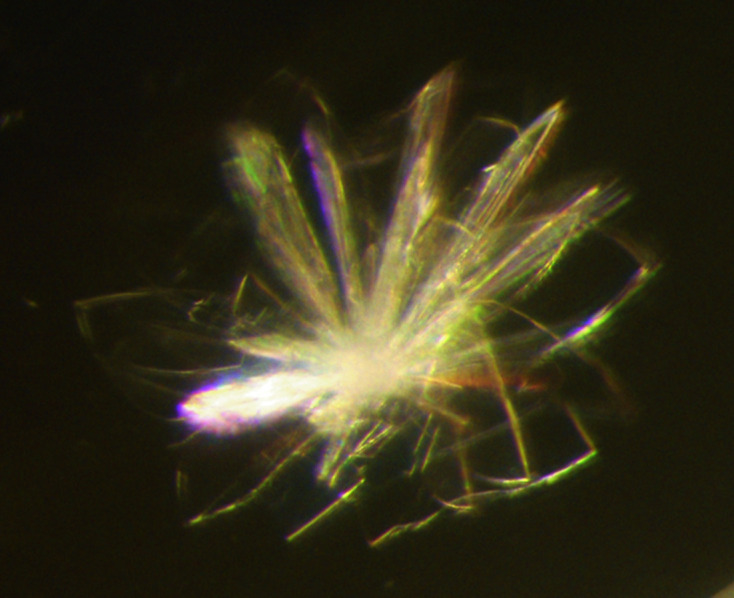
A small cluster consisting of many 3CL M^pro^ crystals with a maximum length of 200 µm. Several clusters were used to generate the seed stocks necessary to nucleate the large single crystal grown for neutron protein crystallography studies at ORNL.

High-throughput crystal screening of 3CL M^pro^ was initially conducted at the Hauptman–Woodward Medical Research Institute (HWI; Luft *et al.*, 2003 ▸) in order to explore new conditions that are capable of growing high-quality crystals for neutron diffraction at room temperature. This identified initial crystallization conditions for further optimization efforts onsite at Oak Ridge National Laboratory (ORNL). 3CL M^pro^ crystals were grown at ORNL using the sitting-drop vapor-diffusion method with a 1:1 protein:well solution ratio. Initial crystal aggregates appeared in 20 µl drops with 25% PEG 3350, 0.1 *M* Bis-Tris pH 6.5 as the reservoir solution using microbridges (Hampton Research, Aliso Viejo, California, USA). Multiple crystal clusters (Fig. 2 ▸) were harvested to generate microseeds with Hampton Research Seed Beads. The microseeding technique provides the reliable reproduction of single crystals, allowing optimization trials for precipitant concentration and pH using grid searching to be carried out (Budayova-Spano *et al.*, 2020 ▸). It is also important to determine whether the freeze–thaw process of concentrated protein samples and microseed stocks has deleterious effects on crystallization. This is especially true for proteases that are capable of autoproteolysis. 3CL M^pro^ samples can grow crystals after storage at −30°C, but preparing aliquots for crystallization avoiding multiple freeze–thawing cycles is highly recommended.

Large-volume sitting-drop vapor diffusion for neutron diffraction-quality crystals of 3CL M^pro^ was performed in 200 µl drops of freshly prepared hydrogenated protein mixed with 18% PEG 3350, 0.1 *M* Bis-Tris pH 6.0, 3% DMSO seeded with 1 µl microseeds (1:500 dilution) in a Hampton Research 9-well plate and sandwich-box setup with 50 ml well solution.

**Figure 3:**
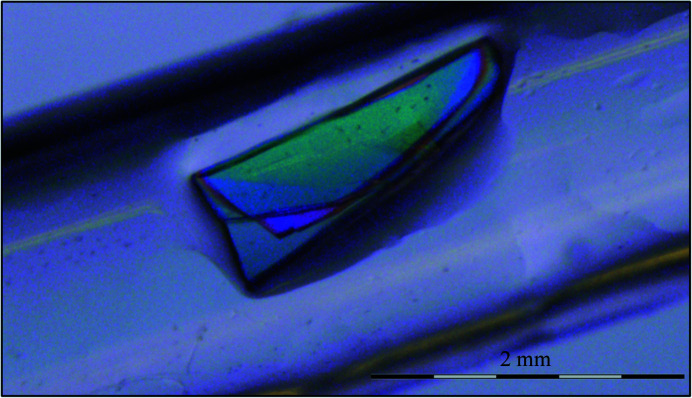
A large protein crystal of 3CL M^pro^ of roughly 2 × 0.8 × 0.2 mm in size, giving a total crystal volume of around 0.3 mm^3^, was used for neutron data collection on the MaNDi instrument at the Spallation Neutron Source.

In this condition, we observed that individual crystals as large as ∼0.8 × 0.2 × 0.05 mm in size appeared if left undisturbed for 3–5 days at 18°C and continued to increase in size for 2–3 weeks until growth ceased. To encourage continued crystal growth, the temperature can be gradually reduced at this point to decrease the solubility of the protein in the crystallization drops. 3CL M^pro^ follows the traditional trend of fewer nucleation sites, microseeds in this case, and a larger crystallization drop volume driving increased crystal size. The largest crystal (Fig. 3 ▸) grew to ∼2 × 0.8 × 0.2 mm (∼0.3 mm^3^) after incubation at 18°C gradually reduced to 10°C over five weeks in a drop with one other crystal. This hydrogenated crystal was then mounted in a capillary with mother liquor prepared with D_2_O and allowed to exchange with deuterium for one week before starting data collection. A pH of 6.6 for the final crystallization drop was measured using a microelectrode, corresponding to a pD of 7.0 (pD = pH + 0.4).

## 3. Results and discussion   

### 3.1. Neutron and X-ray room-temperature data collection   

**Figure 4:**
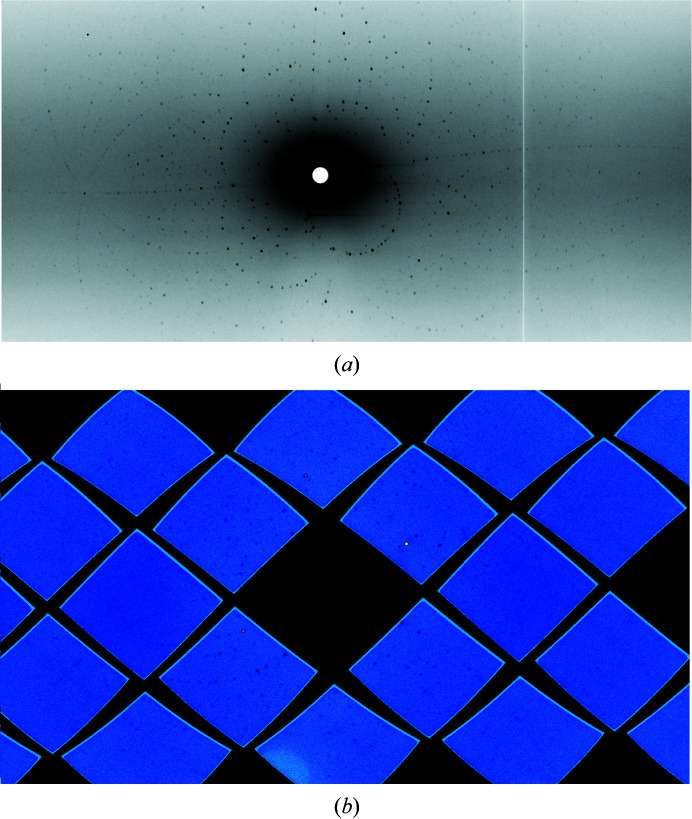
Neutron diffraction images obtained from a 3CL M^pro^ crystal. (*a*) A 12 h exposure on the IMAGINE instrument using all neutrons between 2.8 and 10 Å wavelength. (*b*) A 24 h exposure on the MaNDi instrument using neutrons between 2 and 4.16 Å wavelength.

Crystals were initially screened for diffraction quality using a broad-bandpass Laue configuration using neutrons from 2.8 to 10 Å wavelength at the IMAGINE instrument (Coates *et al.*, 2018 ▸; Schröder *et al.*, 2018 ▸) at the High Flux Isotope Reactor (HFIR) at Oak Ridge National Laboratory (Fig. 4 ▸ *a*). The observed strong neutron diffraction indicated that the crystal was single and worthy of complete data collection. Neutron diffraction data were then collected using the time-of-flight (TOF; Langan *et al.*, 2008 ▸) technique on the Macromolecular Neutron Diffractometer (MaNDi) instrument (Coates *et al.*, 2010 ▸, 2015 ▸, 2018 ▸) at the Spallation Neutron Source (SNS) because the geometry of the MaNDi diffracto­meter provides better reciprocal-space coverage for the low-symmetry monoclinic space group. The crystal was held stationary at room temperature, and diffraction data (Fig. 4 ▸ *b*) were collected for 24 h using all neutrons between 2 and 4.16 Å wavelength. Following this, the crystal was rotated by Δφ = 10° and a subsequent data frame was collected. A total of 23 data frames were collected to form the final neutron data set. The diffraction data were reduced using the *Mantid* package (Arnold *et al.*, 2014 ▸), with integration being performed by three-dimensional TOF profile fitting (Sullivan *et al.*, 2018 ▸). Wavelength-normalization of the Laue data was performed using *LAUENORM* from the *LAUEGEN* suite (Campbell *et al.*, 1998 ▸; Helliwell *et al.*, 1989 ▸).

Using the same crystal, X-ray diffraction images were recorded at ORNL using a Rigaku MicroMax-007 HF X-ray generator coupled with a Dectris EIGER R 4M detector and were reduced using the *CrysAlis^Pro^* software suite (Rigaku, The Woodlands, Texas, USA). The diffraction data were then scaled using *AIMLESS* (Evans & Murshudov, 2013 ▸) from the *CCP*4 suite (Cowtan *et al.*, 2011 ▸; Winn *et al.*, 2011 ▸). The neutron and X-ray data-collection and processing statistics are given in Table 1 ▸.

The resolution of the diffraction data that we obtained with neutrons was limited by the size and the morphology of the hydrogenated crystal that could be produced in a limited time frame. It is envisioned that larger crystal volumes and deuterium labeling of the protein by expression in deuterated medium will allow us to collect neutron data sets to higher resolutions.

## Figures and Tables

**Table 1:** Room-temperature neutron and X-ray data-collection statistics

	X-ray	Neutron
Unit-cell parameters (Å, °)	*a* = 114.93, *b* = 54.69, *c* = 45.22, α = 90, β = 101.46, γ = 90	
Space group	*C*2	
Wavelength(s) (Å)	1.54	2–4.16
No. of unique reflections	12092 (1155)	7995 (765)
Resolution range (Å)	28.16–2.30 (2.38–2.30)	14.95–2.50 (2.59–2.50)
Multiplicity	4.30 (3.70)	5.67 (4.68)
〈*I*/σ(*I*)〉	8.6 (1.7)	13.0 (3.70)
CC_1/2_	0.993 (0.480)	0.961 (0.551)
*R*_p.i.m._	0.069 (0.544)	0.078 (0.156)
Data completeness (%)	98.20 (96.60)	83.93 (83.33)
